# Social Resources for Transplanted Children and Families in European Union Hospitals of ERN TransplantChild

**DOI:** 10.3390/children8090723

**Published:** 2021-08-24

**Authors:** María Jesús Pascau, Laura Pruneda, Ilaria de Barbieri, Matilde Correia, Belén López, Erika Guijarro, Gonzalo Sofío, Esteban Frauca Remacha, Paloma Jara Vega, on behalf of ERN TransplantChild Healthcare Working Group

**Affiliations:** 1 ERN-Transplantchild, La Paz Institute of Biomedical Research, IdiPAZ, Hospital Universitario La Paz, 28046 Madrid, Spain; laura.pruneda@transplantchild.eu (L.P.); belenlopezal24@gmail.com (B.L.); erika.guijarro@gmail.com (E.G.); gonzalo.sofio@transplantchild.eu (G.S.); esteban.frauca@salud.madrid.org (E.F.R.); paloma.jara@transplantchild.eu (P.J.V.); 2 Azienda Ospedaliera di Padova, 35128 Padova, Italy; ilaria.debarbieri@aopd.veneto.it; 3 Centro Hospitalar e Universitário de Coimbra, 30-075 Coimbra, Portugal; matildecorreia@gmail.com

**Keywords:** paediatric transplant, social support, children with special health care needs, long-term care, rare diseases, chronic health conditions, socioeconomic factors, ERN TransplantChild

## Abstract

Social well-being is an intrinsic part of the current concept of health. In the context of chronic disease, there are many challenges we face in order to provide social well-being to patients and their families, even more if we talk about rare diseases. TransplantChild, a European Reference Network (ERN) in paediatric transplantation, works to improve the quality of life of transplanted children. It is not possible to improve the quality of life if the human and material resources are not available. With this study, we want to identify the economic aids, facilities, services, and financed products that are offered to families in different European centres. We also want to find out who provides these resources and the accessibility to them. We designed an ad hoc survey using the EU Survey software tool. The survey was sent to representatives of the 26 ERN members. In this article we present the results obtained in relation to two of the aspects analysed: long-term financial assistance and drugs, pharmaceuticals and medical devices. Some resources are equally available in all participating centres but there are significant differences in others, such as education aids or parapharmacy product financing. A local analysis of these differences is necessary to find feasible solutions for equal opportunities for all transplanted children in Europe. The experience of centres that already provide certain solutions successfully may facilitate the implementation of these solutions in other hospitals.

## 1. Introduction

The definition of health, established by the World Health Organization (WHO) in 1948 [1], in the preamble of its Constitution, states: “Health is a state of complete physical, mental and social well-being and not merely the absence of disease or infirmity”. More than 70 years later, it is still valid, not only because of its provenance but also because of the relevance of some of the concepts contained. Many states take political measures to improve the living conditions of their citizens following WHO recommendations, based on this concept of health. In the present study, we focus on social well-being as a fundamental element of health and analyse some of the elements that may be essential to offer transplanted children and their families a situation of social well-being.

All transplanted children replace their original disease with a life-long chronic condition or disease, mostly imposed by the necessary treatment regimens as immunosuppression to avoid rejection, requiring proper monitoring and handling of post-transplant complications [2].

These life-long chronic conditions or diseases, when they appear in childhood, affect the normal development of the child or adolescent, alter his or her social relationships and, sometimes, can entail a variable degree of physical or psychological incapacity. In the case of transplantation, this incapacity is mostly mild or even non-existent. However, the mere fact of being dependent on medical control and some type of medication, or the alteration of routines with intercurrent events, loss of school hours, hospital admissions, etc., usually affects the child’s daily life. Moreover, in adolescence, the person may perceive these limitations as an inability to keep up with his or her peers. Chronicity is a persistent worry for the family, leading to disruption of the parents’ work, additional expenses, and concern for the future [3].

The fact that these diseases are usually incurable means that they cannot be traditionally treated but must be managed globally. According to the Chronic Disease Management initiative [4], health care can be provided more effectively and efficiently if patients with chronic diseases take an active role in their own care. To face this challenge is not possible if they do not receive support from the necessary resources and experienced professionals, especially those interested in collaborating with their patients so that they are the ones who handle their disease in the best possible way. Patient’s ability to follow medical recommendations, the adaptation of their lifestyles, and access to support resources are factors that influence the optimal management of chronic disease. However, the main issue is that it is impossible to access resources if one is not aware of their existence.

This reality leads to the question of who is responsible for providing the patient and their family with the useful, updated, complete, and timely information on the resources available to include the determinants of the disease into their lives in the least “traumatic” way possible.

Some chronic diseases, such as paediatric transplantation, also belong to the group of rare diseases (RDs), i.e., conditions with a prevalence of less than 5 cases per 10,000 inhabitants, also including criteria of severity and chronicity, which affect the daily lives of around 30 million European Union (EU) citizens [5,6,7]. Their low frequency means that these RDs are poorly understood by professionals and require a great effort on the part of families, due to the limitations and lack of resources in the system to attend to these pathologies. Most cases occur in children, although the prevalence is higher in adults due to the early mortality of some serious childhood diseases and the influence on rates of certain diseases that occur later in life, resulting in a different cumulative prevalence depending on the condition [6,8].

An interdisciplinary approach is required to address RDs, with special efforts aimed at improving the quality of life and the socioeconomic potential of affected individuals and families [9,10]. This effort will not succeed if the unmet social needs of this population are not identified [11]. In the field of social and health care resources, there is a tendency to analyse psychological and social needs together [12,13,14], ignoring the exploration of strictly material resources (including direct financial aid, services or funded resources) that allow families to face the added expenses derived from the care of the child such as medications, hospital stays, adapted leisure, or special school needs.

Social support for children and families with chronic/RDs is the subject of several studies denouncing their psychological, economic, and social vulnerability [15,16,17]. If the concept of “children and youth with special health care needs” [18] is added, the number of articles is even higher. The burden of chronic health conditions on family dynamics or the lack of support resources, in particular those at an acceptable psychological and social cost, has been analysed [19,20,21,22,23,24,25,26], but no article describes the resources that already exist or how they work, an essential step to make specific proposals for improvement.

Resources often exist and are available [27,28], but information needs to be systematic, simple, and structured, and ‘paperwork’ procedures should be simplified. Being part of the ERN TransplantChild is an opportunity to claim these resources at the European level [29], whose support allows us to make an appeal to the institutions. Coordination is needed to ensure access to the minimum resources for any transplanted child and their family.

The main aim of the ERN TransplantChild is to draw up a proposal for the minimum social resources that can be accessed by any family with a transplanted child anywhere in the European Union. Our approach is to identify those resources already in place before, during, and after transplantation; their degree of coverage in each centre/country; and their accessibility, including ease of access, information received [27], and resource providers.

**Figure 1:**
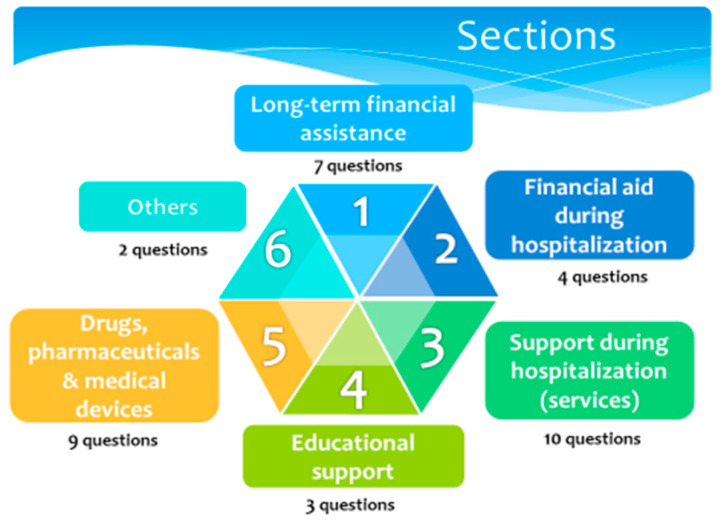
Structure of the Social Resource Survey. General content of the six sections and number of items.

Due to the wide range and large differences between the social resources analysed, in this article, we only present the results of two of the areas studied: (1) long-term financial assistance and (5) drugs, pharmaceuticals and medical devices. The analysis of other areas (Figure 1) will be presented in another article.

## 2. Materials and Methods

### 2.1. Participants

The multidisciplinary team identified two target populations, which face the problem from two different points of view:

1. Families—who have a transplanted child or a child in the process of being transplanted and deal with the demands of that care every day.
2. Social workers—who have an updated and global vision of the available resources.

The picture of the reality that the social workers could offer us may be more complete and up to date, while the picture presented by the families could be incomplete in terms of the resources available. They might not know or might not have used some of them or they might have changed since the moment of transplantation. However, the families were much closer to the reality of the difficulties of the administrative procedures, the waiting times, and the real scope of the aid offered or the failures in the process of information about these resources.

At the time of the study (2020), the ERN TransplantChild was made up of 26 centres, 18 full members and 8 affiliated partners, belonging to 15 different EU countries. There are currently 23 patient associations collaborating with the ERN.

Representatives of all centres and all patient associations were consulted for this study.

On 6 August 2020, an e-mail was sent with the link to the survey. The email provided a brief explanation of the objectives of the study along with instructions on how to complete it.

The e-mail was sent to the representatives of:

- All the hospitals participating in the ERN TransplantChild, requesting them to forward it to the social workers of their centres.
- The patient associations, asking them, in addition to answering the survey, to forward the e-mail to the associated families.

The data collection period was from 6 August 2020 to 25 April 2021. Representatives received weekly reminder mailings.

### 2.2. Measures

An initial team, formed by a variety of professionals (physician, nurse, psychologist, biomedical engineer, and economist) designed a first draft survey. This proposal included all those aspects related to the care of a transplanted child that require some type of institutional support to be carried out. This support can sometimes be economic but, in most cases, the demand responds to the need for material (medicines, devices, sanitary material…), educational, administrative (information and help to apply for different aids), professional (psychological support, physiotherapy, dentist, etc.) or logistic support (temporary accommodation, transport, maintenance, etc.) [30].

The team in charge of designing the survey held two meetings with four social workers who contributed to their professional approach. The social workers provided very valuable information on how to access some of these resources and the bureaucratic difficulties that families encounter when applying for these aids. Their professional vision was of great help in defining the different sections and structuring the survey.

Two virtual meetings were also held due to the COVID-19 pandemic with the representatives of four patient associations: ASION (HSCT), HEPA (Liver), NUPA (Bowel and Multivisceral) and ALCER (Kidney) [31,32,33,34]. The representatives of the transplanted children and their families gave their opinion on the proposed questions, modifying some of them and adding new ones. Aspects that had not been considered initially and that were of concern to the families were incorporated.

After these meetings, and with all the contributions received, the final survey included six sections: (1) long-term financial assistance, (2) financial aid during hospitalization, (3) support during hospitalization (services), (4) educational support, (5) drugs, pharmaceuticals and medical devices, and (6) others (Figure 1).

It consists of 35 general questions to which, in the case of affirmative answers, additional, specific questions were added about the type of aid or service, the ease of access to it, who provides the information about its existence, and when.

As mentioned above, in this article we analyse only the results obtained in sections (1) long-term financial assistance and (5) drugs, pharmaceuticals and medical devices. Table 1 and Table 2 show all the questions included in both sections.

The survey was designed using the EU Survey software tool. The EU Survey [35] is an online survey management system for creating and publishing forms available to the public, e.g., user satisfaction surveys and public consultations. Launched in 2013, the EU Survey is the European Commission’s official survey management tool.

### 2.3. Data Analysis

The data obtained through the EU Survey was dumped into an Excel file and then analysed with an open-access software: Data Studio version 2021 [36]. For the presentation of data results, a descriptive statistical analysis (absolute and relative frequencies) was considered, and Data Studio graphics were generated.

When the research team received several answers from the same centre, the responses obtained were compared to look for divergences. If differences appeared, the research team contacted the social worker of the corresponding centre to clarify the reasons for these differences and the responses were unified into a single response per participant centre.

For calculation of the relative frequencies, all centres with valid answers were considered. In the case of missing answers, information was requested again from the healthcare provider before excluding them from the analysis.

For Section 5 of the survey, one centre was excluded as it did not respond to any of the questions.

## 3. Results

### 3.1. Study Population

A total of 36 responses to the survey were obtained, representing 18 different health care providers. In 15 cases only one response was received per centre, coming from the hospital social worker while from the three remaining centres several answers were received thanks to the response of some families. In these cases, we compared the answers of the social worker and the families, looking for divergences. The information obtained was contrasted with the centre’s social worker, unifying the answers, except for one case, where it was not possible to contact a person responsible for the centre who could clarify the data. Thus, the answers of this centre have been excluded from the sample, making a total of 17 centres entered for the analysis. These 17 health care providers represent 14 European countries: Belgium, Denmark, Estonia, France, Germany, Italy, Latvia, Lithuania, Poland, Portugal, Spain, Sweden, the Netherlands, and the United Kingdom.

### 3.2. Data

#### 3.2.1. Long-Term Financial Assistance

The questions included in this section (Table 1) explore the types of long-term support, both financial and funded services, that families receive to facilitate the care of the transplanted child.

Families receive some type of financial support for the care of the transplanted child in 11 centres (64.70%). In four of these 11 centres, more than one form of this type of assistance is offered, while in the remaining 7 only one. “Payments associated with services (personal assistance, home care, family respite, etc.)” and “Monthly remunerations” are the most common options. The non-existence of this type of aid was reported by 6 centres (35.29%).

**Figure 2:**
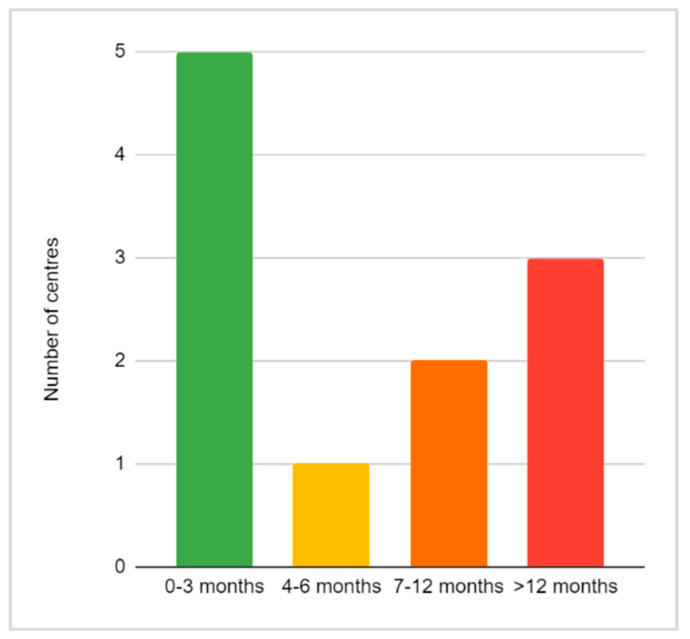
Period from date of application to arrival of aid (0–3, 4–6, 7–12, >12 months).

Regarding the time it takes to receive the aid, families of five centres receive this aid in less than three months from the date of application, in another centre, the aid arrives in the first six months, in two others it can take between 7 and 12 months and for three of them more than a year to arrive (Figure 2).

Another way to help parents care for a sick child is through employee benefits for childcare. Twelve health care providers reported the existence of this type of aid (70.58%), and five (29.41%) did not. Remunerated options (“Paid work leave”, *n* = 9, or “Caregiver leave”, *n* = 8) surpassed unpaid options (“Unpaid work leave: reduction of working hours, leave of absence or similar”, *n* = 5). Both options coexist in the offer provided to parents in four centres. Information on these resources is mostly offered by the hospital (*n* = 10), as compared to social services (*n* = 6) or patient associations (*n* = 5) and primary care (*n* = 5).

The recovery period after the donation is considered as sick leave for living donors in 15 of the 17 centres.

All 100% of the health care providers answered affirmatively to the existence of economic aid for non-pharmacological treatments (hearing aid, eyeglasses, prosthesis, etc.). Funding for these treatments is partial in 10 centres (58.82%). Although 10 centres considered access to this type of aid to be easy or very easy, four centres considered it moderately difficult to access and three centres difficult to access.

**Figure 3:**
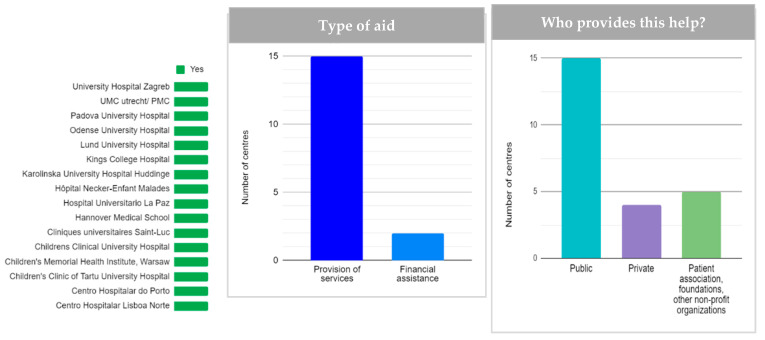
Economic aid for professional treatments (physiotherapy, speech therapy, early stimulation, psychological support, etc.). From left to right, availability, type of aid, and provider of these aids (*n =* 15).

In the context of aid for professional physiotherapy, speech therapy, early stimulation, or psychological support treatments, all centres reported that these are available to families. A total of 15 centres (88.23%) offer the services of professionals directly, with the public sector being the main provider. These public services coexist with those offered by patients’ associations or private providers (Figure 3). The ease of access is also uneven: eight centres rated it as easy or very easy, while nine centres found access moderately difficult or difficult.

Affirmative responses were reduced to 7 (35.29%) and 10 centres (58.82%) when asked about the existence of aids for accessing educational centres and support for complementary activities, respectively (Table 3).

#### 3.2.2. Drugs, Pharmaceuticals and Medical Devices

One of the healthcare providers did not respond to any of the questions in this section, thus, 16 centres were represented in the responses of this section of the survey.

The questions included in this section (Table 2) explore the financing and ease of access to different types of drugs, pharmaceuticals and medical devices.

**Figure 4:**
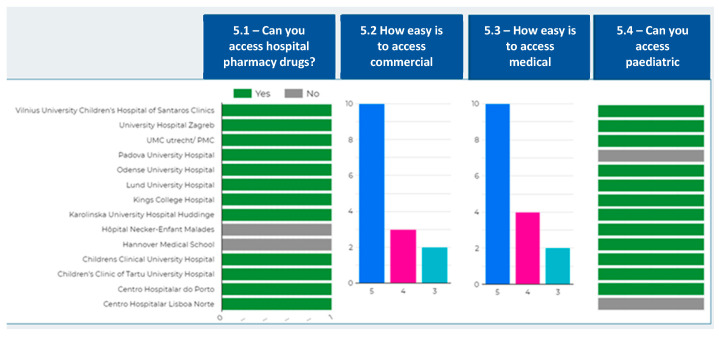
Accessibility to medicines. From left to right, hospital pharmacy drugs, commercial pharmacy drugs, medical equipment, and paediatric doses. For 5.2 and 5.3, *Y*-axis, number of responses; *X*-axis, Likert scale (3 = acceptable, 4 = easy, 5 = very easy).

Immunosuppressants and other drugs frequently used in children undergoing transplantation are subject to special control. In most countries, this means that, although they are sold in conventional pharmacies, families must go through administrative procedures before they can obtain authorization from the health authorities. We asked about the ease of access to commercial pharmacy drugs to identify how complex these procedures could be for families. Fourteen centres (87.5%) rated accessibility to be easy or very easy; however, for 2 centres (12.5%) it was only acceptable (Figure 4).

We also asked about the ease of access to medicines from the hospital pharmacy since some of these restricted drugs are often used on an outpatient basis in transplanted children. Ensuring the supply of these drugs is a matter of concern for families who live far from a hospital or who, living in regions other than the transplant centre, have to arrange for their supply at a hospital other than the one treating them. In 14 centres (87.5%), patients can obtain access to hospital drugs to continue treatment at home if necessary; only two centres (12.5%) reported that they do not have this possibility. Among the 14 centres that provide these drugs, 12 (85.7%) rated the ease of access as easy or very easy and two (14.3%) as acceptable. Access to medical equipment (syringes, masks, gloves, etc.) was included in this section, as preparation and administration of immunosuppressants must be carried out with appropriate protection due to their hazardous nature. On the other hand, medical equipment for the home management of a device (e.g., drains, catheters, feeding pumps for supplemental nocturnal enteral nutrition) is required on a temporary basis by some children. Its access is reported to be easy or very easy by 14 centres (87.5%). These medical devices are mainly provided by loan (12 centres, 85.71%, Table 4).

A specific question on paediatric dosing was included, as some of the required drugs are marketed in adult formats, and do not usually include oral solutions. Moreover, as they are dangerous drugs, it is not advisable to handle them to fractionate or crush them for dilution. It is necessary to resort to magistral formulas of drugs not marketed in liquid formats to be able to dose them properly. Magistral formulas and/or fractionation by a qualified pharmacy service provides safety and guarantees for the preparation and administration of drugs. In 14 centres (87.5%) these practices by the pharmacy services are common, while two centres report not having this service.

**Figure 5:**
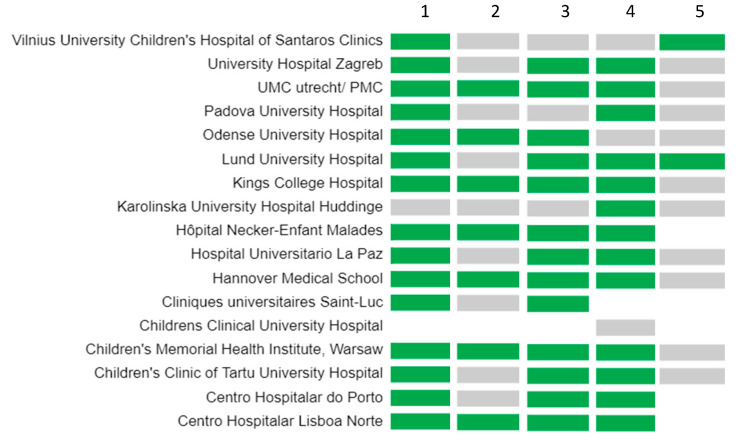
Funding of drugs, pharmaceutical and medical devices in participating centres. From left to right, (1) commercial pharmacy drugs, (2) pharmacy/parapharmacy products, (3) milk or nutritional products (4) medical devices, (5) other drugs. In green, yes; in grey, no; in white, not reported.

In addition to accessibility, we asked about the financing of all these products. Almost all (*n* = 15, 93.75%) confirmed that commercial drugs are financed as a service included in public health care. However, the percentages of financing decreased for other pharmaceutical products or medical devices (Figure 5).

Transplanted children may require, for prolonged or lifelong periods, the use of drugs not included in the health system (due to lack of sufficient evidence of their beneficial effects or the high risk of side effects, among others). However, it is these unfunded drugs (without a therapeutic alternative) that receive the least support (18.75%, Table 4).

Dermatological, oral, and other care products are used to minimize the side effects of long-term immunosuppressant treatment. In more than half of the centres surveyed, these products are not funded (*n* = 9, 56.25%) (Figure 5) and only half of those that do cover the full cost (Table 4).

Children transplanted for congenital malformations or metabolic disorders associated with severe nutritional restrictions need both special/adapted milk formulas and nutritional supplements to regain or maintain an adequate nutritional status. Fortunately, the total cost is covered by the health system in most centres (*n* = 10, 76.92%), but only for all products in two centres (Table 4).

It is worth mentioning that the questions asking to specify the percentage of financing (when the “partial financing” option is selected) were left unanswered in most cases, being obviously unable to offer results in this regard.

## 4. Discussion

### 4.1. Long-Term Financial Assistance

International concern [37,38] about the dimensions that chronic disease (CD) health care is reaching is evident. The exorbitant cost of CDs, in particular the high price of drugs, adds to the limitations that disease causes in all areas of life.

In Europe, initiatives, such as the European Observatory on Health Systems and Policies [38] or the European Chronic Disease Alliance (ECDA) [39], are succeeding each other in order “to reverse the alarming rise in chronic diseases”, but all these initiatives focus their efforts on the political level, observing and analysing the situation, preparing reports, or formulating specific policy recommendations [40].

International, European, and national initiatives are also very numerous in this field. The European Commission [41] states: “Efficient and effective action for rare diseases depends on a coherent overall strategy for rare diseases mobilizing scarce and scattered resources in an integrated and well-recognized way, and integrated into a common effort”. In this 10-page document, exactly six lines are devoted to “specialized social services”, although their essential role in improving the quality of life of people living with a rare disease (RD) is recognized.

As part of this concern for RDs, there are specific initiatives for transplantation financed by the EU to improve the quality of life and achieve common regulations for the rights of living donors. Their recommendations include “Sick leave with 100% payment and socio-medical and protective support if sick leave is prolonged” [42]. The work of all these organizations [43,44,45] and initiatives is reflected in recommendations, manifestos, reports [46], etc., but little or no information appears on specific projects that have been implemented with groups affected by these diseases. Among these initiatives, and specifically focused on paediatric transplantation, is the ERN TransplantChild, promoter of this needed study about resources available.

As far as our study is concerned, the centres reporting the existence of any type of economic aid for the care of a transplanted minor are slightly more than half. That means that many families in Europe face the expenses derived from the care of a transplanted child without any type of economic aid or free service to help the informal support network available to them.

When such support is available, the most common options are payments associated with personal assistance, home care or family respite, or monthly remunerations. It is interesting to note the clarifications of some centres that underline that these payments are not associated with the transplant itself, but with the child’s dependency status or disability. It is also interesting to note that, in the two countries represented in the study by more than one centre, the responses to the question on such aids are opposite. Further research would be needed, but this situation highlights differences in health care for these children even at the regional level. The ideal of equal opportunities for all patients regardless of their place of residence in the field of social welfare proposed by the ERNs is far from being a reality [47].

On the other hand, these long-term benefits should reach families early before the costs overwhelm them. In only five centres do families receive these grants within three months of application. Delays of more than a year should not be acceptable. Surprisingly, the evaluation of the accessibility of these aids in the four centres mentioning delays of more than 12 months are mostly favorable. It is possible that the respondents understood the question to be directed at the difficulty of application procedures and did not consider waiting time as a factor.

Regarding information for families about resources and their processing, our survey only asked about the informant—social services, primary care, hospitals, and patients associations are equally responsible for providing information.

Sick leave for the living donor is today a reality in 15 out of 17 centres. Although it should be a reality in the EU as a whole, it is encouraging to note that in recent years paid sick leave has become a common practice.

A good starting point is there are “economic aids for non-pharmacological treatments” and “aids for professional treatments” in all responding centres because these services are considered to be part of the health care that these children should receive. Accessibility could be improved in many centres, although in almost 60% this was rated as easy or very easy. The role of patients’ associations as providers of these types of services is significant, although not necessary if the professional treatments (offered by the health system in 100% of the centres) covered the needs of children and families. It would be interesting to go deeper into the perceived needs of children and families. Positive numbers may not reflect a high-quality service, or this may not meet the demand [12]. On the other hand, it is noteworthy that treatments are partially funded in 10 centres (58.82%), meaning part of these services is funded by families, and also that six centres consider access to them to be moderately difficult or difficult.

Aids for accessing educational centres are only reported by seven centres. Like the financial aid, these ones are not particularly linked to transplantation, as only two centres stated that transplanted children have preferential access to scholarships.

Support for complementary activities, including camps, extracurricular activities, school support, or leisure is offered in 10 centres, provided in most of them by patient associations, foundations and other non-profit organizations (*n* = 7), i.e., families do not usually find this resource covered by the public system.

The results presented do not respond to the dissatisfaction expressed by families with regard to the social support received or the low scores reported in the quality-of-life questionnaires [48,49]. Obviously, our results are only a first approximation of the problem. A qualitative approach to family experiences is needed: concrete repercussions of the transplantation on the family economy, support resources that are lacking to improve their daily lives, and the role of the health system in improving these situations. Patients’ associations, being first-hand knowledgeable about needs and existing resources, should be an essential partner in designing and implementing any programme aimed at improving the health and social support of transplanted children and their families.

### 4.2. Drugs, Pharmaceuticals and Medical Devices

There is no doubt that pharmaceutical expenditure is one of the most important items for health systems around the world. In an attempt to contain this expenditure, EU countries draw up lists of drugs included and excluded from public financing based on EMA recommendations [50] and control protocols for the supply to avoid indiscriminate use [51]. A significant proportion of drugs commonly used after transplantation, such as immunosuppressants or antivirals, are part of these restricted-use drugs, which obliges families to go through administrative procedures before dispensing them.

Despite this, the results of the survey show that the accessibility to medicines seems to be mostly fulfilled. The different centres/countries have found standardized procedures that make it easier for families to obtain these drugs. In addition, there is also a high level of satisfaction with access to drugs for hospital use, paediatric doses, and medical equipment. The finding that paediatric doses are resolved in most of the centres provides sufficient arguments for the two centres in our study that lack this service to demand its implementation, taking as a reference the models already implemented and with a good level of acceptance.

There is more disparity between centres in terms of the financing of drugs and medical devices, especially according to the type of resource.

Medicines for routine use, marketed in retail pharmacies (commercial drugs), are financed in practically all centres, except one. Moreover, this financing is mostly full, and when it is partial, coverage percentages are above 75%. This means that families are covered by most of the treatment (immunosuppressants, antivirals, antibiotics, corticoids, etc.), which is essential to guarantee equal access to transplantation for all patients, especially when treatment is expensive and should be maintained over the long term to ensure graft survival [52,53]. A similar situation is found with respect to the financing or lending of medical devices, with the majority opting for loans.

The high cost and high consumption of dietary foods, especially in infants whose dietary basis are adapted milk formulas, means that their financing has a strong impact on family economies. Although this financing is mostly assumed by National Health Systems, in three centres it is not, with the consequent economic burden for the families.

The situation with regard to drugs not included in the health system is just the opposite: only three centres reported funding for these products. Funding for pharmacy/parapharmacy products for skin and mucosal care is also uneven.

Lack of funding can have a considerable impact on children’s quality of life. It could mean non-adherence to vitamin or mineral supplements, or to health recommendations on skincare and sun protection or gum and oral mucosa care, as economic constraints may force families to restrict spending on products that may be dispensable compared to other basic needs. In the long term, it can lead to vitamin or mineral deficiencies, cancerous or pre-cancerous skin lesions, or health-threatening infections. Non-adherence results in a much higher healthcare cost than preventive measures to avoid these complications [54]. Several countries have opted for a middle ground, funding these products only for certain pathologies or specific indications. This could be a viable alternative for transplanted children. As we have seen, the procedures to access restricted medicines or products do not seem to pose a major problem for families, who are experts and widely acquainted with the established procedures.

The low response rate obtained in the questions on percentages highlights the lack of information that professionals often have about the resources available to families. It is very difficult to provide adequate information about a resource if complete information about it is not available.

## 5. Conclusions

Knowing the coverage degree of some of the socio-health needs of families with transplanted children does not solve the whole problem but it allows us to identify the starting point and establish measures to strengthen the available resources and facilitate the implementation of those that are absent.

Although socioeconomic situation influences, it is not the main determinant of health. The approach to social resources enjoyed by transplanted children and their families is only the basis on which to work on other social health determinants, such as informal support networks, peer relationships, school/work success, future expectations, autonomy in care, or self-confidence and self-assurance.

A great deal of work must be performed on all these determinants, but none of them can be addressed if the person does not feel that their basic needs, which are highly conditioned by the chronic situation, are covered. Quality of life depends on prevention, but this is not possible without guaranteeing families the coverage of the necessary resources so that the care of the transplanted child does not overwhelm the family economy.

A broad associative movement has developed around the complex issue of RDs in different EU countries, which has played an important role in the development of actions aimed at improving care and research, focusing part of its work on meeting the information needs of affected people/families. The 23 patient associations currently collaborating with the ERN TransplantChild should be the main interlocutors in the decision-making on the resources to be made available and the priority carriers in the transmission of information to families.

The involvement of associations does not exempt health professionals from the responsibility to assess the psychosocial situation when identifying the patients’ needs and to update their knowledge of the health and social resources available for transplanted children and their families and to transmit this information to them.

Further studies are needed to delve into the barriers to accessing these resources, as well as a qualitative focus on the experience of families to obtain additional information about the unmet needs [11] of children with chronic conditions and their families.

## Figures and Tables

**Table 1:** Long-term financial assistance of the Social Resource Survey. Main (numbered) and additional questions and answers are shown.

1.1—Do you receive any type of economic aid for the care of a transplanted minor?	Yes/No
Type of financial benefit	- Monthly remuneration - Payments associated with services (personal assistance, home care, family respite, etc.)—Specify services - Others
Approximate amount of the benefit	€ received per patient
Specify another type of financial aid different from the previous ones	
Period from application to receipt of aid	0–3 months/4–6 months/7–12 months/more than 12 months
Information on this help is accessible through:	- Social services - Hospital - Primary care - Patient associations, foundations, other non-profit organizations
Ease of access	Likert Scale 1 (hard access) to 5 (easy access)
1.2—Do you receive any employee benefit for child care?	Yes/No
Type of benefit	- Paid work leave - Unpaid work leave (reduction of working hours, leave of absence or similar) - Caregiver leave - Other (Specify)
Information on this help accessible through	- Social services - Hospital - Primary care - Patient associations, foundations, other non-profit organizations
1.3—Is work leave available for living donors during recovery period after donation?	Yes/No
Information on this help accessible through	- Social services - Hospital - Primary care - Patient associations, foundations, other non-profit organizations
1.4—Are there economic aids for non-pharmacological treatments? (hearing aid, eyeglasses, prosthesis, etc.)	Yes/No
Type of financing	Total/Partial
Percentage of aid	0–25/25–50/50–75/75–100%
Information on this help accessible through	- Social services - Hospital - Primary care - Patient associations, foundations, other non-profit organizations
Ease of access this help	Likert Scale 1 (hard access) to 5 (easy access)
1.5—Are there aids for professional treatments? (Physiotherapy, speech therapy, early stimulation, psychological support, etc.)	Yes/No
Type of aid	- Financial assistance - Provision of services
Who provides this help?	- Public - Private - Patient associations, foundations, other non-profit organizations
Information on this help accessible through	- Social services - Hospital - Primary care - Patient associations, foundations, other non-profit organizations
Ease of access this help	Likert Scale 1 (hard access) to 5 (easy access)
1.6—Are there aids for accessing educational centres?	Yes/No
Do transplanted children have a preferential access to scholarships?	Yes/No
Type of educational aid	- Education Scholarship - Food Scholarship - Transportation Scholarship - Other (specify)
At what educational stages is this aid offered?	- Children’s education - Compulsory education - Higher education
Amount (in €) of the scholarship	Euros received per patient
Information on this help accessible through	- Social services - Hospital - Primary care - Patient associations, foundations, other non-profit organizations
Ease of access this help	Likert Scale 1 (hard access) to 5 (easy access)
1.7—Are there aids for complementary activities? (camps, extracurricular activities, school support, leisure)	Yes/No
Who provides this help?	- Public - Private - Patient associations, foundations, other non-profit organizations
Information on this help accessible through	- Social services - Hospital - Primary care - Patient associations, foundations, other non-profit organizations
Ease of access this help	Likert Scale 1 (hard access) to 5 (easy access)

**Table 2:** Questions of Section 5—Drugs, pharmaceuticals and medical devices of the Social Resource Survey.

5.1—Can you access hospital pharmacy drugs?	Yes/No
Ease of access	Likert Scale 1 (hard access) to 5 (easy access)
5.2—How easy is it to access commercial pharmacy drugs?	Likert Scale 1 to 5
5.3—How easy is it to access medical equipment (syringes, masks, gloves, etc.)	Likert Scale 1 to 5
5.4—Can you access paediatric doses and master formulas?	Yes/No
Ease of access	Likert Scale 1 to 5
5.5—Are the drugs funded?	All drugs/some drugs/None
Type of funding	Total/Partial
Specify aid percentage	0–25/25–50/50–75/75–100%
5.6—Are other pharmacy/parapharmacy products funded (dermatological, oral care products, etc.)	All products/Some products/None
Type of funding	Total/Partial
Specify aid percentage	0–25/25–50/50–75/75–100%
5.7—Is the milk or nutritional products funded?	All products/Some products/None
Type of funding	Total/Partial
Specify aid percentage	0–25/25–50/50–75/75–100%
5.8—Is there funding/loaning of medical devices? (monitor, pulse oximeter, feeding pump…)	Loaned/Funded/None
Type of funding	Total/Partial
Specify aid percentage	0–25/25–50/50–75/75–100%
5.9—Do you receive aid for drugs not included in the health system	
Type of funding	Total/Partial
Specify aid percentage	0–25/25–50/50–75/75–100%

**Table 3:** Accessibility to long-term financial assistance. *n =* number of centres where it is available. In bold, main questions.

Aids for Accessing Educational Centres (*n* = 7, 35.29%)	
	*n* (%)
Preferential access to scholarships Yes	2 (11.76%)
Type of educational aid Education scholarships Transportation scholarships Food scholarships Scholarships for material	7 7 7 7
Educational stage Children’s education Compulsory education Higher education	7 7 2
Main provider Social services	7
Ease of access (only 6 responses) Easy or very easy Moderately difficult or difficult	5 1
**Support for complementary activities (camps, extracurricular activities, school support, leisure, etc.) (*n* = 10, 58.82%)**	
Main provider Patient associations, foundations, and other non-profit organizations	7
Ease of access Very easy Easy Moderately difficult Very difficult	1 4 4 1

**Table 4:** Funding of medicines at participating centres (number of centres). N/A = not applied, N/R = not reported.

	Commercial Pharmacy Drugs	Pharmacy/Para-Pharmacy Products (Dermatological, Oral Care, etc.)	Milk or Nutritional Products	Medical Devices (Monitor, Pulse Oximeter, Feeding Pump…)	Other Drugs Not Included in the Health System
Funding of products					
Yes No N/R	15 (93.75%) 1 1	7 (43.75%) 9 1	13 (81.25%) 2 1	14 (87.5%) 2 1	3 (18.75%) 10 5
Number of products/drugs					
All Some None	8 7 1	3 4 9	2 11 2	N/A N/A N/A	2 1 10
Provision of funding					
Loaned Funded None	N/A N/A N/A	N/A N/A N/A	N/A N/A N/A	12 2 2	N/A N/A N/A
Type of funding					
Total Partial	11 4	3 4	10 3	9 3	1 2
Aid percentage					
0–25% 26–50% 51–75% 76–100%	0 0 1 2	N/R N/R N/R N/R	N/R N/R N/R N/R	N/R N/R N/R N/R	N/R N/R N/R N/R

## Data Availability

The data presented in this study are available on request from the corresponding author. The data are not publicly available due to some personal information was gathered. An archived dataset generated during the study can be found here: https://datastudio.google.com/u/0/reporting/f445516d-b758-4dad-9bb4-764ed33f963f/page/fLopB (accessed on 10 May 2021).

## Hospitals:

**Center**	**City**	**Country**
Hôpital Necker-Enfant Malades	Paris	FR
Hannover Medical School	Hannover	DE
Children’s Memorial Health Institute, Warsaw	Warsaw	PO
Vilnius University Children’s Hospital of Santaros Clinics	Vilnius	LT
Karolinska University Hospital Huddinge	Huddinge	SE
Children’s Clinical University Hospital	Riga	LV
Children’s Clinic of Tartu University Hospital	Tartu	EE
Odense University Hospital	Odense	DK
University Medical Center/Princess Maxima Center	Utrecht	NL
University Hospital Zagreb	Zagreb	HR
Lund University Hospital	Lund	SE
Hospital Universitario La Paz	Madrid	ES
Saint-Luc Hospital	Brussels	BE
King’s College Hospital	London	UK
Azienda Ospedale Università Padova	Padova	IT
Centro Hospitalar Universitário do Porto (Porto)	Porto	PT
Centro Hospitalar e Universitário de Coimbra	Coimbra	PT
Centro Hospitalar e Universitário de Lisboa	Lisboa	PT

## Social Workers

To Elena Aparicio Plaza, Amaia Isabel Martín, Marta Sánchez Deusa and Ana de Vicente Fer-nández for their kindly contribution in reviewing the questionnaires.
